# Direct Acoustic Stimulation at the Lateral Canal: An Alternative Route to the Inner Ear?

**DOI:** 10.1371/journal.pone.0160819

**Published:** 2016-08-08

**Authors:** Nicolas Verhaert, Joris Walraevens, Christian Desloovere, Jan Wouters, Jean-Marc Gérard

**Affiliations:** 1 KU Leuven—University of Leuven, Department of Neurosciences, ExpORL, Leuven, Belgium; 2 Department of Otolaryngology, Head and Neck Surgery, University Hospitals Leuven, Leuven, Belgium; 3 Cochlear Technology Centre Belgium, Mechelen, Belgium; 4 Department of ENT and Head and Neck Surgery, Cleveland Clinic Abu Dhabi, Abu Dhabi, United Arabic Emirates; University of Windsor, CANADA

## Abstract

Severe to profound mixed hearing loss is associated with hearing rehabilitation difficulties. Recently, promising results for speech understanding were obtained with a direct acoustic cochlear implant (DACI). The surgical implantation of a DACI with standard coupling through a stapedotomy can however be regarded as challenging. Therefore, in this experimental study, the feasibility of direct acoustic stimulation was investigated at an anatomically and surgically more accessible inner ear site. DACI stimulation of the *intact*, *blue-lined* and *opened* lateral semicircular canal (LC) was investigated and compared with *standard oval window* (OW) coupling. Additionally, stapes footplate fixation was induced. Round window (RW) velocity, as a measure of the performance of the device and its coupling efficiency, was determined in fresh-frozen human cadaver heads. Using single point laser Doppler vibrometry, RW velocity could reliably be measured in low and middle frequency range, and equivalent sound pressure level (L_*E*_) output was calculated. Results for the different conditions obtained in five heads were analyzed in subsequent frequency ranges. Comparing the difference in RW membrane velocity showed higher L_E_ in the *LC opened* condition [mean: 103 equivalent dB SPL], than in *LC intact* or *blue-lined* conditions [63 and 74 equivalent dB SPL, respectively]. No difference was observed between the LC opened and the standard OW condition. Inducing stapes fixation, however, led to a difference in the low frequency range of L_*E*_ compared to LC opened. In conclusion, this feasibility study showed promising results for direct acoustic stimulation at this specific anatomically and surgically more accessible inner ear site. Future studies are needed to address the impact of LC stimulation on cochlear micromechanics and on the vestibular system like dizziness and risks of hearing loss.

## Introduction

In the past decade, considerable temporal bone and human clinical research has been conducted on the efficiency of acoustic hearing implants, such as active middle ear implants (AMEIs) and direct acoustic cochlear implants (DACI). Because these implants are indicated for various pathologies, many different ways of implantation have been described, each with its own advantages and disadvantages [1–7]. Recently, a systematic review of clinical results by Verhaert et al. concluded to a certain degree of variability in functional outcome partially related to patient selection and coupling techniques [8].

For severe to profound mixed hearing loss, only powerful implants can produce sufficient output to stimulate the remaining cochlear reserve. A Codacs™ DACI system produces high output, thereby achieving encouraging speech perception results, even compared to the best conventional treatments, such as hearing aids with or without stapedotomy surgery [9,10]. The surgical procedure is often regarded as challenging, as it involves posterior tympanotomy, fixation of the transducer inside a small mastoid cavity, and then a transmastoid or endaural approach for stapedotomy with coupling of a stapes prosthesis to an actuator [11]. Taking into account its initial phase, the average time of surgery was around 3.5 hours (communication from the manufacturer) and the approach carries risks of facial nerve exposure or damage to residual hearing, well known from cochlear implant surgery and stapes surgery in case of advanced otosclerosis or tympanosclerosis [12]. Straightforward, reproducible acoustic stimulation of an anatomically easy accessible inner ear site could reduce these risks associated with middle and inner ear implant surgery. Also, future cochlear implant devices may include a separate offset acoustical stimulation, away from the cochlea. The lateral semicircular canal is considered a stable landmark during mastoidectomy with little anatomical variation. The canal is approached easily when drilling away the cortical bone of the mastoid and the juxtaposed mastoidal cells.

Driving the cochlea at other sites, such as a ‘third-window’, has been proposed experimentally in an animal model [13] and implemented in selected clinical cases, where neither the oval window nor round window (RW) could be reached [14]. In procedures for semicircular canal plugging, Parnes et al. already reported hearing preservation 20 years ago [15]. In an animal study, Smouha and Inouye reported stable hearing thresholds [16] after the transection of the lateral semicircular canal. In a preliminary study in cats [17], stimulation on the superior semicircular canal with a piezoelectric vibrator was investigated in comparison to cochlear stimulation. The authors reported that the cochlear microphonic frequency response was similar in case of vibration at the oval window or at the superior canal fenestra. In the same paper, it was described that a patient underwent a temporary placement of the vibrator at the superior canal under local anesthesia, showing improved hearing with neither vertigo nor observable nystagmus during stimulation [17].

The aim of this experimental study is (1) to investigate the feasibility of acoustic stimulation of the lateral semicircular canal (LC) in the low and middle frequency hearing range and (2) to assess coupling efficiency compared to the standard oval window coupling [11]. This experimental study is limited to a cadaveric evaluation and further feasibility studies still need to be performed before clinical application of LC stimulation can be considered, above all because of the risks of hearing loss and dizziness when opening the labyrinth.

## Methods

### 2.1 Temporal bone preparation

Fresh-frozen whole heads with intact temporal bones and no history of ear disease were evaluated after obtaining authorization to use organs and tissues for research (Science Care, Inc., Phoenix, AZ, USA, www.sciencecare.com). The medical history of each head was provided by the supplying company. After thawing, all experiments were performed within an 8-hour period and the heads were rinsed meticulously and largely sealed in a plastic bag to prevent mechanical changes due to dehydration during the experiments similar to other authors [18,19]. No specimens were refrozen. Microscopic visual inspection was carried out to verify temporal bone integrity and quality, as well as the absence of otologic disease, as recommended in the ASTM F2504-05 standard practice [20]. The study was approved by the medical ethics committee of University Hospitals Leuven.

Surgical preparation consisted of a canal wall-up mastoidectomy, with preservation of the posterior border of the mastoid cavity for placement of the implant’s fixation system. The facial recess was opened through a large posterior tympanotomy to achieve exposure of the stapes crura, stapes footplate and the round window membrane (RWM), with removal of its secondary mucosal membrane. Care was taken not to alter ossicular chain integrity. Visual inspection was performed to check for perilymph leakage. One head, considered for training purpose, was excluded from further analysis, as drilling of the LC accidently led to perilymph evaporation. Since then, both the canalotomy and stapedectomy, respectively, were strictly performed under saline fluid to avoid air bubbles. The heads were firmly held in a soft holding block during all procedures.

### 2.2 Acoustic hearing implant

Throughout this study, the driving force for acoustic stimulation was an electromagnetic actuator, which is part of an acoustic hearing implant, the Codacs™ DACI system (Cochlear™ Ltd. Sydney, Australia). The surgical procedure to implant the DACI system was previously described in detail [11]. In brief, following the manufacturer’s specifications, the actuator’s artificial incus (with 1 mm diameter), also named rod, is connected to a conventional stapes prosthesis coupled to the perilymph of the inner ear at the level of the oval window niche. Coupling to the inner ear is done after removal of the stapes superstructure, i.e. stapedotomy or stapedectomy. For clinical application, a calibrated-hole technique using a laser or skeeter burr is preferred for stapedotomy. In cadaver head experiments, stapedectomy with complete removal of the entire footplate and placement of fibrous tissue for oval window sealing is more feasible. This is consistent with the first DACI implantations, as described by Häusler et al. [3]. For 7 out 8 heads, both steps were performed under fluid to prevent air from entering during the cadaver experiments; the sealing was kept moist during the measurements. Previous investigations have confirmed linearity of actuator output up to 1 V root mean square (RMS) [21].

### 2.3 Measurement setup

An insert earphone (ER-2, Etymotic Research, USA) was fixed inside the external ear canal with adaptive foam to seal it externally. The tube of a probe microphone (ER-7C, Etymotic Research, USA) was positioned within 1–2 mm of the tympanic membrane and used as a reference. The insert earphone assembly was calibrated before each use. The earphone was driven by a sine sweep at approximately 94 dB SPL between 100 and 10000 Hz generated by an audio analyzer (UPV, Rhode and Schwartz, Munich, Germany). For velocity measurements, a Zeiss microscope-mounted laser Doppler vibrometry (LDV) system (OFV5000 Vibrometer Controller, OFV-534 Compact Sensor Head and A-HLV MM 30 Micromanipulator; Polytec GmbH, Waldbronn, Germany) was utilized. To enhance light reflection, markers made from small pieces of reflective tape (0.4 mm^2^) were placed on the posterior stapes crus and RWM. Anatomical preparation ensured an optimal laser angle of about 70–80° to the posterior crus of the stapes and maximum 45° to the RWM. This meant a limited amount of drilling away the overhanging lip at the round window niche was performed if needed. During analysis a cosine correction to the measurement data was applied using the visually estimated angle. For the DACI acoustical stimulation, being presented at the LC or at the oval window (OW), a forward stimulation pathway [22] can be hypothesized and therefore measuring the velocity at the RWM was preferred to the stapes footplate displacement. Importantly, for air and bone conduction, the RWM moves in a complex and variable manner at frequencies above 1.5 kHz, certainly above 3 kHz, but independent of the acoustic stimulation level in the range between 80 and 110 dB SPL [23,24]. Therefore vibration measurements at a single position cannot be used as indicator of absolute RWM volume displacement. Other studies have showed that the vibrations measured at the center of the RWM increase linearly with acoustical stimulation level in the range of 50 to 110 dB SPL [25]. As mentioned by Grossöhmichen et al. [26], the constancy of the vibration pattern allows a relative estimation of stimulation efficiency in forward stimulation, moreover for the comparison of different coupling situations. Hence, similarly [26] the position of the reflective tape on the RWM was kept constant approximately at an anterior-central position of the RWM throughout each of the experiments. The LDV controller was set to 10 mm/s/V with a 100 kHz low-pass and 100 Hz high-pass filter. Stapes and RW velocity were evaluated with closed-field acoustic measurements in a quiet room. The noise floor of the measurement set-up and room was assessed by measuring the response without any stimulation present. Responses with an SNR smaller than 5 dB were excluded from analysis.

An additional experiment on the vibratory output pattern of the RWM was performed at multiple positions of the RWM in one head. The aim was to confirm the validity of the single-point measurements at the RWM relevant for our study. This was performed in preparation of future possibly more precise intracochlear or even intralabyrinthine pressure measurements. In this experiment, the investigated conditions as detailed in section 2.5 were similar, but the velocities of the stapes footplate and the RWM were measured at the four vertices of a rectangular piece of reflective tapes placed in the center of the RWM and between the stapes crura, in accordance to the measurement method developed by Gostian et al [27]. The integration of four points allows a confirmation of the single point LDV measurements comparing conditions detailed in section 2.5 relative to each other rather than the absolute output. In addition, the phase was measured to assess the effect of the canalotomy. The volume velocities at a specific frequency were considered to be valid only if all four velocity measurements were valid concerning the SNR. The three-dimensional movements were calculated at the vertices of the reflective tape, similar to the work of Stenfelt et al. and Gostian et al. [23,27]. The reflective tape was considered a rigid body. The relative positions of the 4 points to the center of the RWM or stapes footplate were used as the coordinates x and y with the velocity expressed as v_pz_ (x, y) = v_oz_ + U_ox_ y—U_oy_ x. The system of equations given by these 4 coordinates is overdetermined and therefore a least squares estimation was used to find a solution. By using the coordinates relative to the center of the RWM or stapes footplate, the center velocity was determined by the values ${\hat{v}}_{oz}$ [27] multiplied by the respective velocities. Each measurement at the vertices was measured twice.

### 2.4 Equivalent sound pressure level determination

Obtained middle ear transfer functions (H_*TV*_) were calculated as V_*U*_/P_*T*_, where V_*U*_ is the stapes velocity (RMS) for acoustic stimulation and P_*T*_ is the sound pressure (RMS) measured at the tympanic membrane [20]. H_*TV*_ was computed in units of dB m/s normalized to acoustic input pressure (Pa).

To quantify the performance of a device coupled to the ossicles, a method calculating equivalent sound pressure levels (L_*E*_), in equivalent dB SPL, and maximum equivalent sound pressure levels (L_*E*,*max*_) from sound-induced stapes velocity was developed by Rosowski et al. [28]. Different to AMEIs coupled to the RW [6], computing the transfer function (H_*EV*_) of the stapes, i.e. the velocity of the stapes relative to the vibration input at the RW, was not possible, as during standard DACI implantation the stapes superstructure is removed and the cochlea is driven by a conventional stapes prosthesis. Therefore, as in the procedure described by Chatzimichalis et al. [29], output was derived from RWM velocity measurements, expressed in dB m/s. In their study measurements were performed with a single point LDV, at a fixed location. L_E_ were calculated from the RWM output [26]. Stimulation of 1 V RMS was applied directly to the actuator and a hypothetical electrical input to the actuator of E_*max*_ = 1V RMS was assumed to determine the L_*E*,*max*_.

$$L_{Emax}=20\;\log_{10}\;(H_{EV}\times E_{max}/2\times 10^{-5}\;Pa)$$ (Eq 1)

The audio analyzer simultaneously captured stapes or RW velocity output from the LDV and probe microphone signals near the tympanic membrane. In general, the recommendations as in the ASTM practice were followed for specimen preparation, measurement setup and analysis.

### 2.5 Experiments

**Fig 1:**
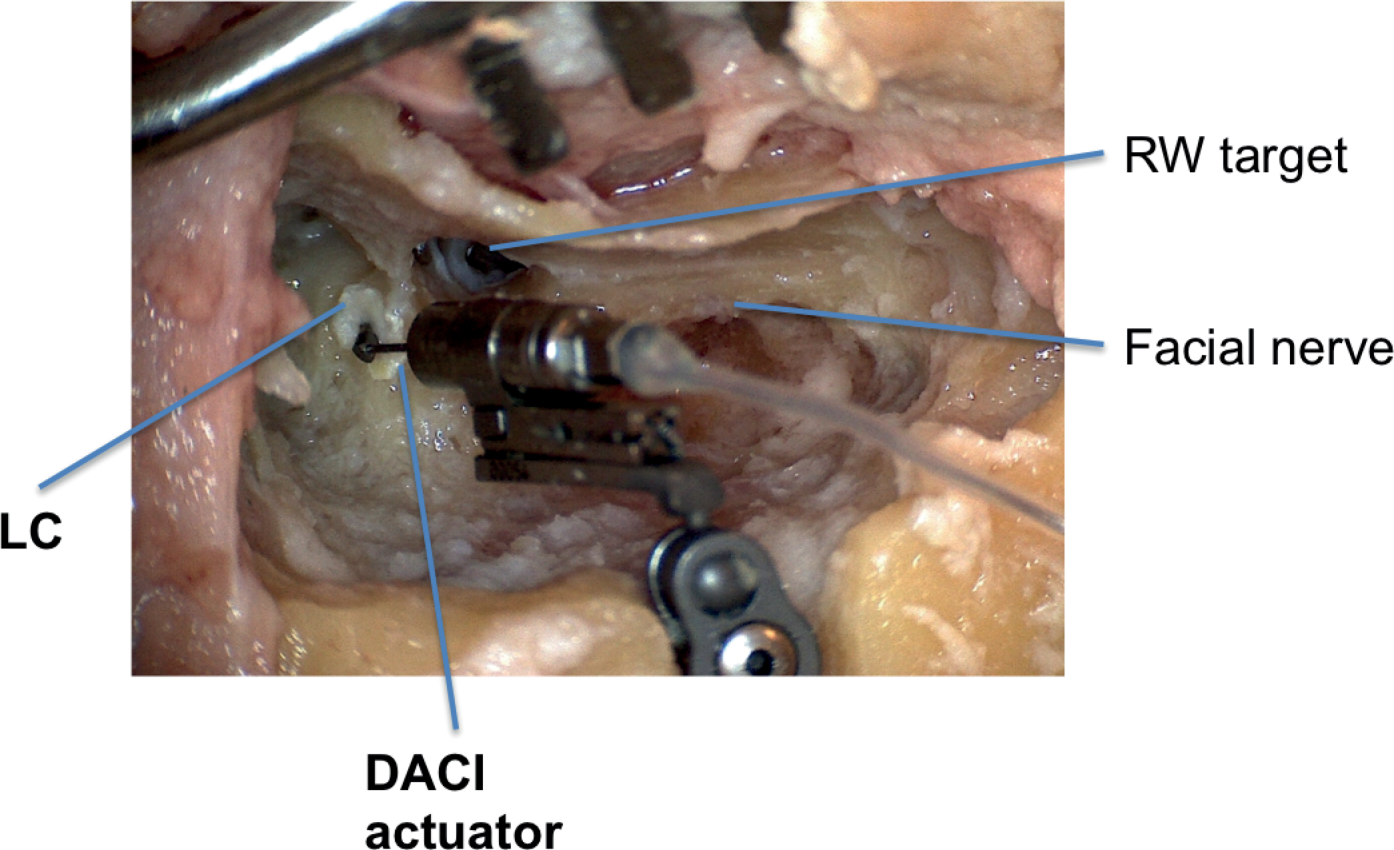
Coupling of DACI actuator with its artificial incus to the lateral semicircular canal (LC). Fresh head preparation showing LC stimulation (*LC opened* condition) with fascia interposition and demonstrating the perpendicular position of the actuator’s tip (diameter of 1 mm) to the LC. The axis of motion was more or less tangent to the axis of lateral canal. View from posterior with reflective tape on RWM. Note the proximity of the facial nerve with the narrow access to the oval window.

Each cadaver head was implanted with a DACI being coupled to the inner ear in 5 consecutive conditions, as illustrated in Fig 1. The same investigators conducted each experiment, in the same sequence, reducing variability. RW velocity was measured at a similar angle for each condition. Three repetitive LDV measurements were carried out for each velocity measurement. Because of the high number of LDV measurements comparing different methods, a single point laser technique at a fixed point was preferred over a scanning laser. Vibration of the prepared head was expected to be low as full heads sealed in plastic bag and soft fixation material was used. In each of the first four conditions, contact between the actuator’s artificial incus and the coupling site was made neither applying too much pressure on the surface nor impeding a correct movement of the tip of the rod, as noted by a shift in the resonance frequency.

Condition 1 –*LC intact*: The actuator was approximated against an intact LC easily accessed through a basic mastoidectomy, keeping the ossicular chain and buttress intact. Contact was made at the dome of the LC, posterior to the ampulla.

Condition 2 –*LC blue-lined*: Using a 0.5-mm diamond burr and intermittent irrigation the LC was blue-lined, indicating imminent appearance of the endosteum of the labyrinth, similar to posterior canal occlusion [15]. Again, the actuator was approximated against the blue-lined surface, without breaching it, at the highest point of the dome of the LC.

Condition 3 –*LC opened*: Under fluid, the canal was opened further (size about 0.3 x 0.5 mm) until the last endosteal bone shell could be gently fractured with a fine spatula, keeping the membranous labyrinth intact. This was defined as a canalotomy. The surgical technique was inspired by the early fenestration operation described by Sourdille and Lempert, although the endosteal flaps were not folded back [30,31]. At this point, no aspiration was performed. The opening, still under fluid, was directly covered with fibrous tissue obtained from the temporalis fascia. The tip of the actuator’s artificial incus was gently brought into contact in a perpendicular manner to the opening (Fig 1). The long rod was mostly in an almost parallel manner to the direction of the lateral canal.

Condition 4 –*LC opened with stapes fixation*: After completing condition 3, stapes footplate fixation was achieved using techniques previously described for the dental acrylic application [18,32]. Thus, LC stimulation could be investigated in case of stapes fixation, similar to otosclerosis pathology, as this is one of the principal clinical indications for DACI implants. The LDV was used to measure stapes velocity attenuation at the stapes posterior crus (3 heads) and/or the RWM (3 heads).

Condition 5 –*Standard oval window (OW)*: After elimination of any dental acrylic surplus, the entire stapes was removed under fluid without suction and fibrous tissue was immediately placed to seal the oval window. The footplate was removed for reasons of reproducibility and to avoid footplate manipulations. The actuator of the DACI implant was then introduced through the opening of the posterior tympanotomy and placed about 4–6 mm above the oval window. A conventional stapes prosthesis, titanium K-Piston type, 0.6 mm in diameter with a loop (Heinz Kurz GmbH, Dusslingen, Germany), was placed on the fibrous tissue in the oval window and firmly crimped to the actuator. The fascia was maintained on the canalotomy to avoid leakage. Carefully, the seal was kept moist to prevent air from entering.

After checking for normality with a Kolmogorov-Smirnov test for every condition, the data was statistically analyzed using repeated-measures analysis of variance (ANOVA) of the main factors *condition* (coupling method) and *frequency* and the difference in RW velocity as a dependent variable with Bonferroni post-hoc analysis. Third octave band frequencies were analyzed represented by the center frequency in the figures. The analyses were performed separately for each frequency range using the geometric mean (low, 0.1–0.8 kHz; middle, 0.801–2.5 kHz; high 2.501–8 kHz), and comparisons were made between the given conditions using paired samples t-tests, with RW velocity as a dependent variable. Similar to Tringali et al, these three frequency ranges were chosen to investigate the coupling in three clinically relevant ranges [[6]. For all analyses, an effect size (*r*) was calculated using Eq 2, derived from the *t*-value and degrees of freedom (d*f*) [33].

$$r=\sqrt{\frac{t^{2}}{t^{2}+df}}$$ (Eq 2)

Comparisons, expressed as the difference in dB of the various RWM velocities for each coupling method with respect to the *standard OW* condition, were calculated for each ear, and then averaged. Finally, the additional effect of stapes fixation on top of the *LC opened* condition was investigated (*LC opened with stapes fixation*).

## Results

### 3.1 Closed-field acoustic transfer functions

**Fig 2:**
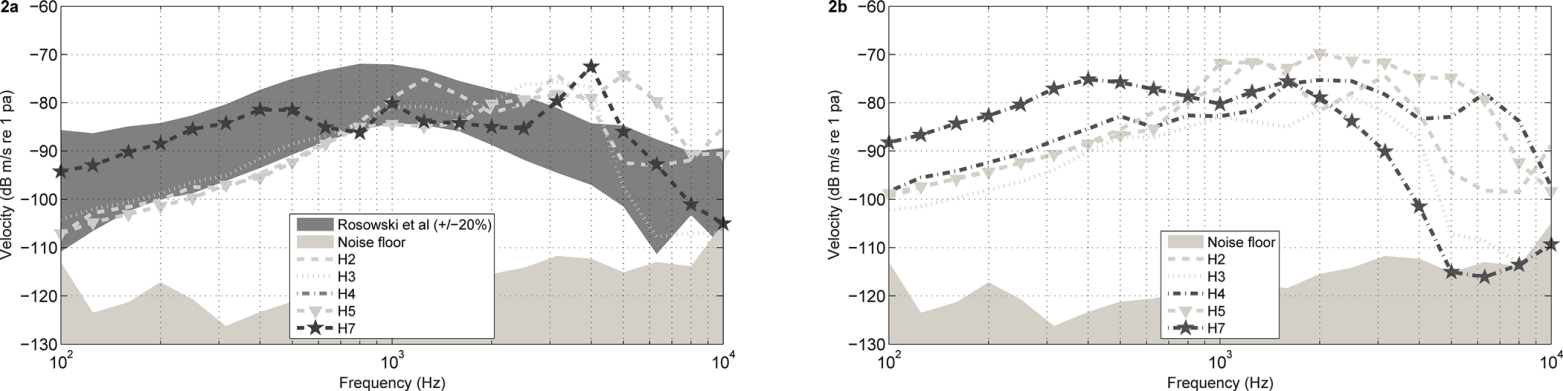
**a,b**. Closed-field acoustic middle ear transfer function (H_*TV*_) at the stapes (**a**) and the RWM (**b**) in 5 human heads (in dB m/s normalized to 1 Pa) with an air conduction stimulus of 94 dB SPL. Additionally in panel a, as a gray shaded region, the mean and +/- 95% CI of H_*TV*_ measured in a large population of temporal bones [28] and the noise floor level are shown.

Fig 2 depicts stapes and RW velocity as individual curves obtained with a sine signal for each cadaver head, plotted against the range, as described by Rosowski et al. [28] for temporal bones. Both stapes and RW transfer functions were comparable. Five of eight temporal bones roughly met the extended inclusion criteria [28] if we take into account that the peaks at 3–4 kHz are due to the closed-ear canal resonance. Even though some heads did not meet the acceptance criteria at every single frequencies, they were included because the main goal in this experimental study was to investigate relative intra-head comparisons for different coupling strategies, as mentioned previously. This is consistent with the wide variability described before in living humans with normal hearing [34].

### 3.2 LC stimulation

**Fig 3:**
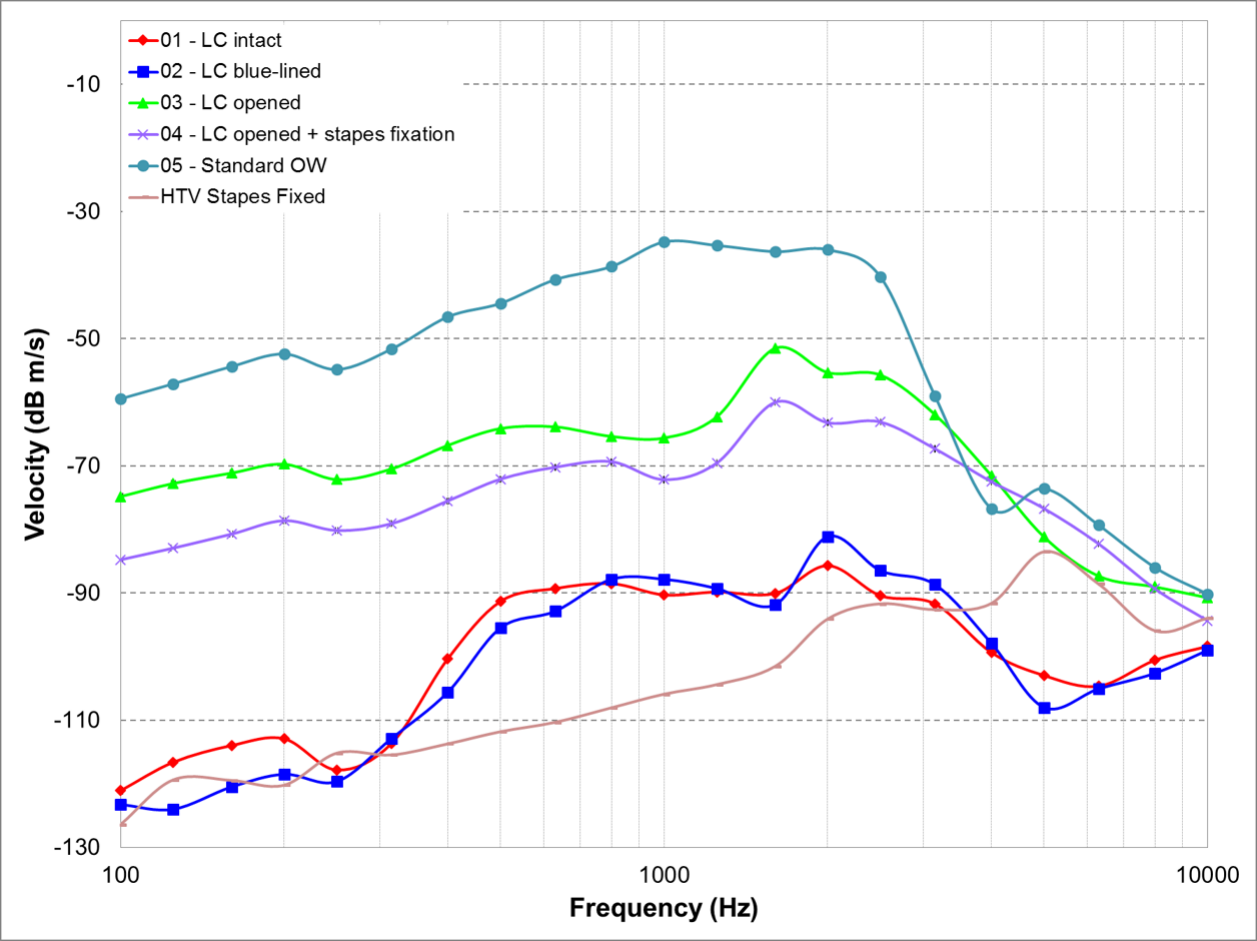
Electrovibrational transfer function the DACI in different coupling conditions, velocity (dB m/s) measured at RWM from one representative specimen (H5) is shown. The stapes velocity (H_*TV*_) of the fixed stapes is also shown.

As described above, the coupling of the actuator to the LC was investigated at 1 V RMS through the output measured at the RWM level in terms of RW velocity in five heads. Measurement variability per condition per head was very low (0.8 dB for 3 repetitions) and therefore the average value of the three consecutive measurements was used. Fig 3 shows the electrovibrational transfer function of one representative head for the different coupling methods. No compensation to the amplitude of the RW velocity at 0.1 kHz was needed. In all coupling conditions, the RW output was similar of shape. *LC intact* and *LC blue-lined* conditions yielded similar outputs, just above noise level), but well below the output in the *standard OW* condition, especially in the low and middle frequency range. In the *LC opened with or without stapes fixation* and in *standard OW* condition, a peak response occurred around 2 kHz, corresponding to the approximate actuator response frequency. For these conditions, up to 6 kHz, a minimum-maximum difference of less than 30dB was noted.

**Fig 4:**
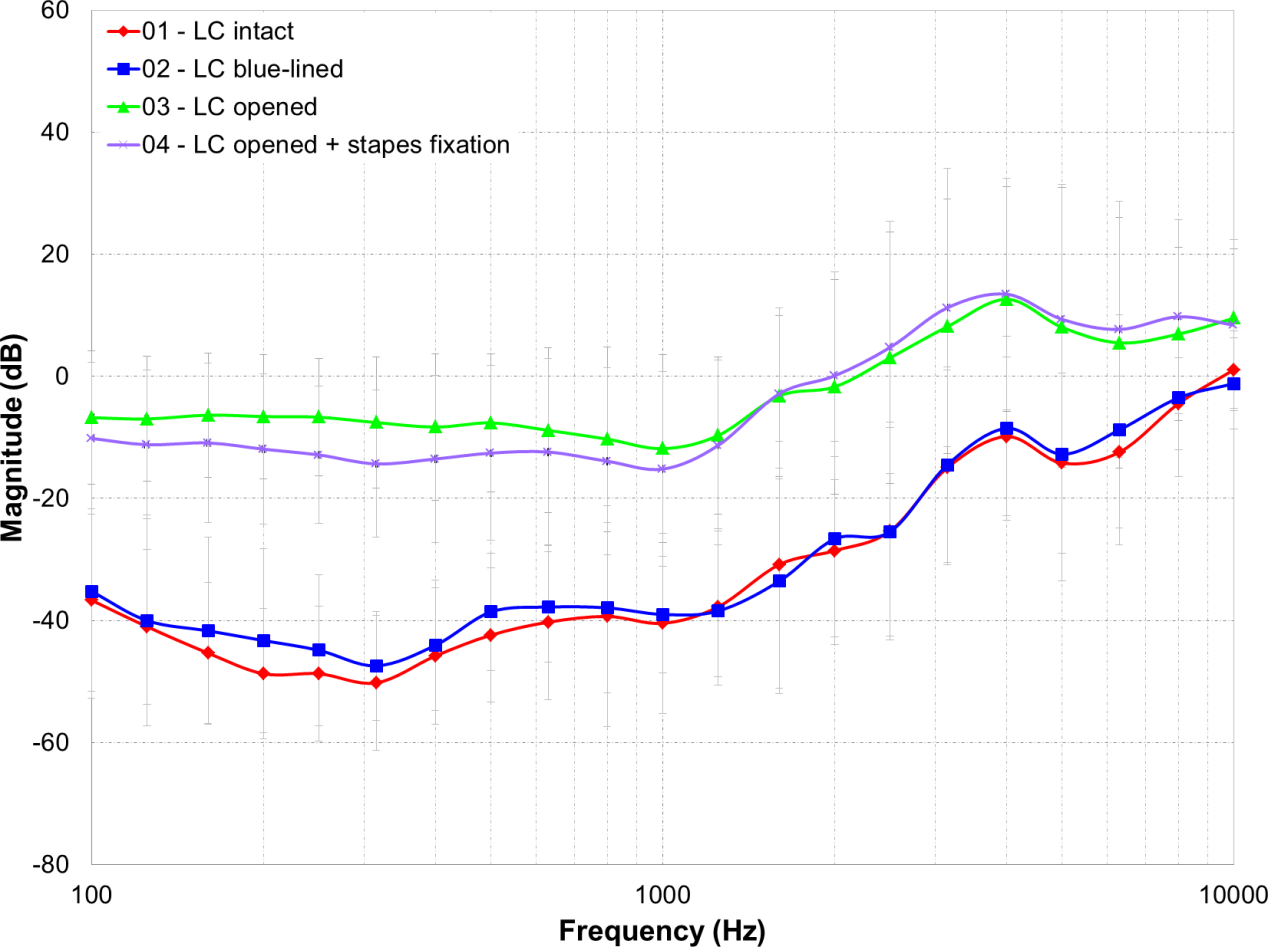
Average of differences in dB of the RW velocity of different coupling methods with respect to the standard OW coupling for each ear (n = 5).

Comparisons, expressed as the difference in dB of the various RWM velocities for each coupling method with respect to the *standard OW* condition, were calculated for each ear, and then averaged, as depicted in Fig 4. Mean differences were largest in the low and middle frequencies for the *LC intact* and *blue-lined* conditions with an average difference over all frequencies of 31.2 dB (SD = 13.1) and 29.7 dB (SD = 13.9), respectively. *LC opened* condition appeared to have an advantage over *LC intact* and *blue-lined* conditions with smaller, non-significant differences, with respect to the *standard OW* coupling. Over all, an average difference of 2.1 dB (SD = 14.8) was noted.

A repeated-measures analysis of the differences was performed. Mauchly’s test indicated that the assumption of sphericity had not been violated. Significant effects were post hoc analyzed using Bonferroni test. There was a significant main effect of condition [*F*_(3,12)_ = 39.99, *p*< 0.001]. Pairwise comparisons reveal that difference in RW velocity was significantly higher in conditions *LC intact* and *LC blue-lined*, than in conditions *LC opened with or without stapes fixation*. The analysis noted a significant main effect of frequency [*F*_(20,80)_ = 11.09, *p* < .001], indicating the impact on the frequency range. There was a significant interaction effect of condition and frequency [*F*_(60,240)_ = 3.46, *p*< 0.001]. Pairwise comparisons with post-hoc Bonferroni corrections, showed a statistical significant difference (*p* < 0.05) between *standard OW* vs *LC intact* and *blue-lined* condition in comparison to the *standard OW* condition vs *LC opened without or with stapes fixation* up to a 1.25 kHz and at 4 and 5 kHz.

Stimulation effectiveness was assessed by calculating the L_*E*._ (n = 5), as detailed in Table 1. With an average equivalent level (eq.) of 62.7 dB SPL (SD = 14.4 dB) in the *LC intact* condition, DACI stimulation did not perform as good compared to the *standard OW* position (average L_*E*,_ of 103.3 eq. dB SPL, SD = 17.0). Blue-lining the LC yielded improved output, reaching 73.6 eq. dB SPL (SD = 11.2 dB). With *LC opened* stimulation, an L_*E*,_ of 103 eq. dB SPL (SD = 15.0 dB) was reached, comparable with the *standard OW* condition.

**Table 1:** Mean Equivalent output levels and standard deviation (SD) for 5 conditions tested at third-octave band frequencies (in response to 1 V_rms_ at the actuator) (n = 5).

	LC intact		LC blue-lined		LC opened		LC opened with stapes fixation		Standard OW	
Frequency (kHz)	Mean [eq dB SPL]	SD [dB]	Mean [eq dB SPL]	SD [dB]	Mean [eq dB SPL]	SD [dB]	Mean [eq dB SPL]	SD [dB]	Mean [eq dB SPL]	SD [dB]
**0.1**	68.6	5.3	73.1	3.9	103.8	14.6	94.5	15.2	107.0	18.6
**0.125**	64.7	5.7	68.7	8.3	104.5	15.6	95.0	15.5	107.9	18.6
**0.16**	59.8	5.8	68.1	8.6	105.2	16.1	96.8	15.1	108.8	18.6
**0.2**	58.3	9.4	67.2	10.7	105.1	16.6	97.6	14.9	109.5	18.4
**0.25**	57.2	8.2	63.1	9.5	103.4	15.5	95.4	13.1	108.0	16.7
**0.315**	54.4	10.3	60.2	13.2	102.9	15.8	94.1	13.7	108.3	17.9
**0.4**	52.3	12.7	64.2	15.7	102.6	17.0	92.2	16.0	107.7	20.5
**0.5**	53.5	12.9	69.8	14.3	101.2	18.2	93.8	13.9	107.4	20.9
**0.63**	55.7	10.1	72.1	16.1	101.6	18.8	97.2	15.1	108.9	22.3
**0.8**	49.3	9.4	69.9	18.6	98.7	19.6	96.0	15.9	107.5	20.4
**1**	50.2	14.5	68.3	10.9	97.4	19.9	94.2	17.8	107.5	17.1
**1.25**	54.3	15.0	68.7	7.4	99.7	18.0	97.1	18.4	107.1	17.9
**1.6**	61.6	22.8	71.8	7.1	105.2	14.3	102.8	17.6	106.0	18.9
**2**	63.0	24.4	76.8	10.2	108.2	12.6	105.2	17.0	105.5	15.7
**2.5**	64.2	22.9	76.9	7.9	111.5	14.7	107.4	20.1	103.2	14.8
**3.15**	66.4	23.0	80.5	10.6	109.6	16.1	106.2	25.6	95.7	9.2
**4**	65.9	16.1	80.2	9.1	102.9	11.4	105.1	18.4	89.3	7.2
**5**	72.4	21.6	82.3	10.7	102.2	9.8	109.9	14.1	96.2	15.7
**6.3**	75.5	20.9	82.8	14.5	98.1	7.9	105.1	11.2	94.2	20.0
**8**	82.5	16.3	89.5	15.2	99.6	8.1	102.0	12.0	92.5	15.4
**10**	86.4	14.2	92.1	13.1	100.5	13.9	100.7	15.5	92.0	12.5
**average**	62.7	14.4	73.6	11.2	103.0	15.0	99.5	16.0	103.3	17.0
**max**	86.4	24.4	92.1	18.6	111.5	19.9	109.9	25.6	109.5	22.3

Calculated L_*E*_ data were normally distributed for all conditions and all frequency ranges. Paired samples t-test showed that L_*E*_ was significantly lower in the *LC intact* condition than the *standard OW* condition in the low (*t*(4) = -5.97, *p* = .004, *r* = .94) and middle (*t*(4) = -2.88, *p* = .045, *r* = .82) and high (*t*(4) = -2.82, *p* = .048, *r* = .82) frequency range, confirming the added value of direct acoustic inner ear stimulation. There was no impact of blue-lining on coupling efficiency (*LC intact* vs. *blue-lined* conditions) in the middle and high frequency ranges (mid: *t*(4) = -1.74, *p* = .16, *r* = .66; high: *t*(4) = -1.20, *p* = .29, *r* = .52). In the low frequency range there was a significant difference (*t*(4) = -5.79, *p* = .004, *r* = .95). When comparing *blue-lined* and *standard OW* conditions, the findings were unsurprisingly similar to the *LC intact* condition. Here, results were significant in the low (*t*(4) = -5.48, *p* = .005, *r* = .94) and middle (*t*(4) = -3.83, *p* = .019, *r* = .88) frequency range, and the same trend, though not significant, was found for the high (*t*(4) = -2.03, *p* = .11, *r* = .71) frequency range.

In the *LC opened* condition, higher RW velocity was obtained than in the *LC intact* condition for all frequency ranges (low: *t*(4) = -9.36, *p* = .001, *r* = .98; middle: *t*(4) = -3.20, *p* = .033, *r* = .85; high: *t*(4) = -2.76, *p* = .049, *r* = .81). The same advantage of the *LC opened* condition was found compared to the *LC blue-lined* condition (low: *t*(4) = -9.78, *p* = .001, *r* = .98; middle: *t*(4) = -5.44, *p* = .006, *r* = .94; high: *t*(4) = -4.32, *p* = .012, *r* = .91).

Both *LC opened* and *standard OW* conditions appeared to have an advantage over *LC intact* and *blue-lined* conditions. There was no indication of any difference in performance of the DACI coupled to the *opened LC* or the *standard OW* (low: *t*(4) = -9.20, *p* = .410, *r* = .41; middle: *t*(4) = .042, *p* = .968, *r* = .02; high: *t*(4) = .932, *p* = .404, *r* = .42).

In a separate head measurement, the RWM three-dimensional movement was calculated from the velocity measurements at the vertices of the reflective tape at the RWM. Here again, mean differences for calculated RW velocity with respect to *standard OW* condition were largest in the low and middle frequencies for the *LC intact* and *blue-lined* conditions with an average difference over all frequencies of 28.6 dB (SD = 21.9) and 27.3 dB (SD = 21.5), respectively. *LC opened* condition appeared to have an advantage over *LC intact* and *blue-lined* conditions with smaller differences with respect to the *standard OW* coupling. The measured RW velocity was within one +/- SD of the mean five heads with single point LDV measurements except for the middle and high frequency range in the *LC opened* condition, showing possibly a suboptimal coupling or more complex RW vibration pattern. A repeated-measures analysis of the differences of all heads (n = 6) did confirm a significant effect of condition (*p* < 0.001) and frequency (*p* < 0.001). The interaction was even so significant [*F*_(60,138)_ = 3.97, *p*< 0.001]. Pairwise comparisons revealed a significant effect between the differences (*p* < 0.05) for *LC intact* and *blue-lined* condition in comparison to the *standard OW* condition vs *LC opened without or with stapes fixation* up to a 1.0 kHz.

Looking at *L*_*E*_ calculations, paired samples t-test showed similar results to the previous analysis with n = 5 above, except in one case. In contrast to the analysis with single point LDV only, L_*E*_ analyses did not reveal a statistically significant difference between *LC intact* condition and *LC opened* for the middle (*t*(4) = -2.5, *p* = .16, *r* = .75) and high (*t*(4) = -2.1, *p* = .042, *r* = .70) frequency range. More data is needed for adequate comparisons between single and multiple point LDV analysis at RWM.

### 3.3 Stapes fixation

**Fig 5:**
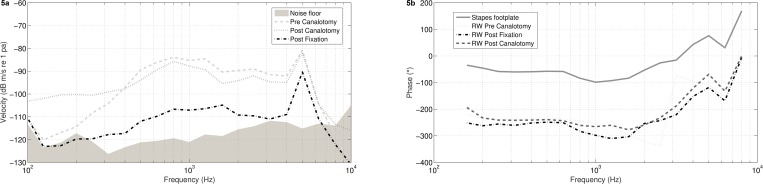
Effect on H_*TV*_ at RW of canalotomy and of experimental induced stapes footplate fixation shown for velocity (a) and for phase (b) in one head.

Stapes footplate fixation resulted in loss of stapes velocity, with an average attenuation of 13 dB (SD = 7 dB) at the stapes (3 heads) and 14 dB (SD = 8 dB) at the RW (3 heads), measured via acoustic stimulation. The paired samples t-test revealed a significant difference in stapes velocity and in RW velocity in the low frequency range (*p <* .03) up to 500 Hz, but not in the middle or high range. The effect of canalotomy, covered by fascia, on the RW output for acoustic stimulation can be appreciated in Fig 5. As shown, phase measurements at the RWM nicely show a 180° shift compared to the stapes footplate displacement up to 2 kHz. After canalotomy, a small difference of about 20° is noted.

The *LC opened with stapes fixation* condition investigated the influence of induced stapes fixation on LC stimulation with the DACI. Fig 4 depicts the calculated difference for this coupling method with respect to the *standard OW* condition, plotted against the other experimental conditions. As mentioned above the repeated-measures analysis of the differences was performed, showing a statistical significant difference between the *standard OW* vs *LC intact* and *blue-lined* condition in comparison to the *standard OW* condition vs *LC opened with stapes fixation*. The paired samples t-test of the calculated L_*E*_ data (Table 1) between the *LC opened* condition and the *LC opened with stapes fixation* condition suggested added value of the latter in the low frequency range (*t*(4) = 3.78, *p* = .019, *r* = .88). L_E_ was similar in the middle (*t*(4) = .57, *p* = .60, *r* = .27) and high (*t*(4) = -1.36, *p* = .25, *r* = .56) frequency range. Similarly to the *LC opened* condition, higher L_*E*_ was found in the *LC opened with stapes fixation* condition compared to the *LC intact* condition (low: *t*(4) = -6.57, *p* = .003, *r* = .95; middle: *t*(4) = -2.72, *p* = .049, *r* = .81; high: *t*(4) = -2.92, *p* = .043, *r* = .82) and *LC blue-lined* condition (low: *t*(4) = -6.55, *p* = .003, *r* = .96; middle: *t*(4) = -3.10, *p* = .036, *r* = .84; high: *t*(4) = -4.26, *p* = .013, *r* = .91). Direct comparison between the *standard OW* condition and the *LC opened with stapes fixation* condition also revealed no difference in terms of performance, similarly to the *LC opened* condition. Including the head with LDV 4 point measurement, revealed similar results except for the middle frequency ranges *comparing LC opened with stapes fixation* to condition *LC intact* and *blue-lined*, here no significant differences were noted. Other comparisons revealed stable and significant results.

## Discussion

### 4.1 LC stimulation

Experimental DACI stimulation at the level of the lateral canal was investigated in different conditions. The inertial mode for bone conduction hearing is the strongest in the lateral direction when the axis of the vibration coincides with the axis of the position of the cochlea [35]. Subsequently, the first two conditions (*LC intact and LC blue-lined*) aimed to evaluate DACI performance without opening the canal, thereby also avoiding risks of hearing loss and vertigo. Similar to stapes surgery, opening the inner ear should be carried out with utmost care in order to avoid drainage of perilymph potentially leading to serious hearing loss and vertigo events. Although not entirely similar, opening and plugging procedures of the semicircular canal have been associated with limited risks like mild hearing loss (2.3% in the study of Agrawal and Parnes [36]), instability or even vertigo [15,37]. In a prospective study with 28 patients undergoing a lateral canal plugging for Menière’s disease, two cases (7%) of labyrinthitis with deafness were noted [38]. In our study, the output measured at the RW was, however, insufficient for amplification in either of these two conditions. The repeated measures analysis of the comparison of the coupling with respect to the *standard OW* condition showed a significant effect of condition, frequency and an interaction effect. L_*E*_ analyses revealed modest but statistically significant added value of the *LC blue-lined* condition over the *LC intact* condition for the low (*p* = .004) frequency range. Once the LC was opened, large-spectrum mean output of 103, 108 and 107 eq. dB SPL in the lower, middle and higher frequency range, respectively, and a peak output of 112 eq. dB SPL were obtained. This performance is slightly below previous temporal bone investigations [39], using 1 V RMS input to an actuator coupled to the RW. With the DACS-PI (Phonak Acoustic Implants SA, Switzerland) coupled to the oval window at an input of 0.3 V RMS, an L_*E*,*max*_ of 110 eq. dB SPL was recorded [29], but higher input voltages were not investigated in this study. As the system acts linear, going from 0.3 V to 1 V RMS would mean an increase of 6 dB SPL. Interestingly, in the current study, no sharp resonance peak was noted in RW velocity with DACI stimulation of the LC, in contrast to standard oval window coupling [40]. In standard oval window coupling with stapes prosthesis, this resonance peak is damped after the healing process in vivo. In addition to increased performance in a wide frequency range, the LC stimulation pathway could potentially facilitate fitting issues, circumventing the need for damping the peak to avoid overstimulation.

As shown, no loss of DACI performance when stimulating the LC was observed when the stapes footplate was fixed. This is in line with findings on third-window stimulation with AMEIs in case of stapes footplate fixation [13]. When comparing *LC opened* condition with the *LC opened with fixed stapes* condition, calculated L_*E*_ output showed a small yet statistically significant difference in the low frequency range after stapes fixation. Accordingly, it may be hypothesized that stimulation of a third-window, in the context of stapes fixation, with an unobstructed round window, results in improved impedance at the base of the cochlea. Therefore, the DACI device can possibly be applied in case of stapes footplate fixations, such as otosclerosis. It should be noted that systematic phase investigations were not taken into account and warrant further study.

Unlike conventional DACI coupling after calibrated-hole stapedotomy, a stapedectomy was performed in the current study, as a surgical laser was not available in the temporal bone laboratory. The absence of a stapes footplate resulted in lower than expected high-frequency amplification, although the results were roughly consistent with RW DACI stimulation and DACI stimulation with a 0.8-mm stapes piston [29,39]. Neither *LC opened* condition, with or without stapes fixation, showed any significant difference compared to the *standard* condition, demonstrating that in terms of performance, LC stimulation after canalotomy with fascia interposition could be an alternative for the *standard* DACI procedure. Compared to an AMEI device coupled to the RW [41], much higher velocity values, i.e. peak values up to -56 dB m/s versus about -90 dB m/s, were obtained with DACI stimulation in both *LC opened* and *standard* OW conditions.

### 4.2 Experiments

This study explores the feasibility of acoustical stimulation at the lateral canal on human cadaver heads. Because of the high number of experimental conditions, at this stage a single point LDV at a fixed position has been used, similar to Grossöhmichen et al. [26]. Similarly, the single point LDV measurement at RWM cannot provide reliable information in high frequency modes [24,42], robust conclusions can only be drawn from the low and middle frequency range. Coupling an actuator to the LC, at the side of the scala vestibuli, possibly creates a kind of forward stimulation with the RWM acting as a pressure outlet [22]. Nevertheless, underestimation of equivalent sound pressure output is possible to some extent. Another point of uncertainty is the influence of the canalotomy. In our study, input impedance of the cochlea does not appear to be changed by DACI application in the *LC opened* condition (though covered with moist fibrous tissue). In the experiments of Pisano et al. [43], patching of the third-window did allow reversible intracochlear pressure measurements. Current results, showed that the difference in phase measurements between stapes footplate and RW, performed in the additional head, was not significantly altered after the canalotomy. Also the RW output in response to acoustic stimulation in the ear canal only dropped with a maximum of 5 dB in the low and middle frequency range, not in the high frequency range. The authors feel, however, that the results should be interpreted with care as stimulation at a third window, though covered, could affect the normal cochlear input impedance and the related difference in pressure between the scala tympani and vestibuli. Although less important for clinical DACI use, [43] showed that at low frequencies, near 100 Hz, the sound induced pressures at the scala vestibule decreased by about 10 dB (with large error bars) for a 0.5 mm dehiscence at the superior semicircular canal. They concluded that in these cases the stapes velocity cannot be fully related to the sound conduction in the cochlea. Therefore, consistent with the view of Stieger et al. [22] the RWM was preferred to measurement of stapes footplate displacement.

A reduction in stapes velocity of at least 30 dB has previously been described [32], and 20 dB for RW velocity, which is more than the respective 13 dB and 14 dB reduction in stapes and RW velocity reported in our study. One reason for the difference might be that fresh-frozen heads are less opted for drying of the dental acrylic. As mentioned before, the canalotomy effect seemed minimal. But the creation of a third-window on the side of the scala vestibuli, in vivo sometimes represented by a conductive hearing loss [44] could have altered the results. The third-window leads to an alternative pressure outlet instead of the pressure difference between the oval and round window [45], so the impact of stapes fixation can be minimized. Further studies are required to investigate the effect of LC canalotomy on the magnitude and phase of the middle ear transfer function, and on cochlear micromechanics. Use of intracochlear and even intralabyrinthine pressure sensors, regardless of experimental alterations such as stapes fixation or stapedectomy, may help to shed light on in this matter [46,47].

### 4.3 Clinical implications and future research

Notwithstanding the experimental data, coupling a powerful acoustic hearing implant to an anatomically easy accessible site can have far-reaching clinical implications. The lateral semicircular canal can be easily approached after performing a basic mastoidectomy.

The described LC stimulation technique derives from closed fenestration procedures introduced at the beginning of the 20^th^ century by Jenkins and Holmgren, and later refined by Sourdille and then by Lempert [48]. In contrast to open fenestration surgery, contact with open air, epidermal growth and granulation tissue is avoided, minimizing risks of vertigo or dizziness, similar to plugging procedures. Furthermore, the size of the canalotomy in our study was only 0.5 mm, and it was covered with fascia. In a study [49] on 20 patients with cholesteatoma-induced lateral canal fistula, postoperative vestibular problems were noted in a small number of subjects: two cases with spontaneous nystagmus and two cases with benign paroxysmal positional vertigo. Future research will clearly need to investigate in e.g. a finite element model and animal study including the mechano-sensitive organs before clinical implementation is started assessing for example, risks of bony tissue regrowth in case of non-stimulation and effects on the vestibular system in the event of non-linearity. It is known that bone conduction implants do not affect the vestibular organ. It can therefore be assumed that acoustic stimulation, with frequencies above 100 Hz, is well above vestibular stimulation frequency range. Nevertheless a mechanical stimulation nearby the ampulla of the lateral canal could potentially evoke vestibular sensations. In a preliminary study [17], an acoustic hearing implant stimulated the fenestrated superior semicircular canal in one patient undergoing a posterior canal procedure. This patient did not complain of hearing loss, vertigo or any motion sensation. A shortcoming in our cadaver study is that possible non-linear effects causing vertigo, cannot be investigated. LC fibrosis could potentially hinder the recorded output. Electrophysiological measurements, first in animal models as demonstrated in AMEIs in case of third-window stimulation [13], then in humans using reliable techniques, can provide objective feedback on adequate coupling to the inner ear, and also on the correct auditory processing in the cochlea and brainstem [21].

Finally, with the development of specially designed drills or robot-controlled microdrill that avoid perforation of the membranous labyrinth or with the use of laser-calibrated hole techniques, results may be reproducible and risks of induced hearing loss well controlled [50].

## Conclusions

This study demonstrates the feasibility of LC stimulation with a DACI device for severe to profound hearing loss. Opening the lateral semicircular canal resulted in performance efficiency similar to conventional oval window coupling. Stapes fixation did not impede DACI performance at the level of the LC. Using a single point LDV technique, the measurements can only be interpreted for low and mid frequencies. Importantly, future studies need to carefully address both the vestibular and long-term effects of LC stimulation and its impact on cochlear micromechanics.

## References

[1] Martin C, Deveze A, Richard C, Lefebvre PP, Decat M, Ibañez LG, et al European results with totally implantable carina placed on the round window: 2-year follow-up. Otol Neurotol 2009;30:1196–203. 10.1097/MAO.0b013e3181c34898 19890224

[2] Baumgartner W-D, Böheim K, Hagen R, Müller J, Lenarz T, Reiss S, et al The vibrant soundbridge for conductive and mixed hearing losses: European multicenter study results. Adv Otorhinolaryngol 2010;69:38–50. 10.1159/000318521 20610913

[3] Häusler R, Stieger C, Bernhard H, Kompis M. A novel implantable hearing system with direct acoustic cochlear stimulation. Audiol Neurootol 2008;13:247–56. 10.1159/000115434 18259077

[4] Schwab B, Salcher RB, Maier H, Kontorinis G. Oval window membrane vibroplasty for direct acoustic cochlear stimulation: treating severe mixed hearing loss in challenging middle ears. Otol Neurotol 2012;33:804–9. 10.1097/MAO.0b013e3182595471 22699990

[5] Luers JC, Hüttenbrink K-B, Zahnert T, Bornitz M, Beutner D. Vibroplasty for mixed and conductive hearing loss. Otol Neurotol 2013;34:1005–12. 10.1097/MAO.0b013e3182990d2b 23820796

[6] Tringali S, Koka K, Deveze A, Holland NJ, Jenkins HA, Tollin DJ. Round window membrane implantation with an active middle ear implant: a study of the effects on the performance of round window exposure and transducer tip diameter in human cadaveric temporal bones. Audiol Neurootol 2010;15:291–302. 10.1159/000283006 20150727 PMC3202919

[7] Verhaert N, Fuchsmann C, Tringali S, Lina-Granade G, Truy E. Strategies of active middle ear implants for hearing rehabilitation in congenital aural atresia. Otol Neurotol 2011;32:639–45. 10.1097/MAO.0b013e318212023c 21436753

[8] Verhaert N, Desloovere C, Wouters J. Acoustic hearing implants for mixed hearing loss: a systematic review. Otol Neurotol 2013;34:1201–9. 10.1097/MAO.0b013e31829ce7d2 23921919

[9] Lenarz T, Verhaert N, Desloovere C, Desmet J, D’hondt C, González JCF, et al A Comparative Study on Speech in Noise Understanding with a Direct Acoustic Cochlear Implant in Subjects with Severe to Profound Mixed Hearing Loss. Audiol Neurootol 2014;19:164–74. 10.1159/000358004 24556905

[10] Zwartenkot JW, Snik AFM, Mylanus EAM, Mulder JJS. Amplification options for patients with mixed hearing loss. Otol Neurotol 2014;35:221–6. 10.1097/MAO.0000000000000258 24448281

[11] Lenarz T, Zwartenkot JW, Stieger C, Schwab B, Mylanus EAM, Caversaccio M, et al Multicenter study with a direct acoustic cochlear implant. Otol Neurotol 2013;34:1215–25. 10.1097/MAO.0b013e318298aa76 23921930

[12] Vincent R, Oates J, Sperling NM. Stapedotomy for tympanosclerotic stapes fixation: is it safe and efficient? A review of 68 cases. Otol Neurotol 2002;23:866–72. 12438848 10.1097/00129492-200211000-00010

[13] Lupo JE, Koka K, Jenkins HA, Tollin DJ. Third-window vibroplasty with an active middle ear implant: assessment of physiologic responses in a model of stapes fixation in Chinchilla lanigera. Otol Neurotol 2012;33:425–31. 10.1097/MAO.0b013e318245cecb 22334156

[14] Pau HW, Just T. Third window vibroplasty: an alternative in surgical treatment of tympanosclerotic obliteration of the oval and round window niche. Otol Neurotol 2010;31:225–7. 10.1097/MAO.0b013e3181cc07fd 20042904

[15] Parnes LS. Update on posterior canal occlusion for benign paroxysmal positional vertigo. Otolaryngol Clin North Am 1996;29:333–42. 8860931

[16] Smouha EE, Inouye M. Partial labyrinthectomy with hearing preservation: Frequency-specific data using tone-burst auditory brain stem response. Otolaryngol—Head Neck Surg 1999;120:146–52. 10.1016/S0194-5998(99)70398-0 9949344

[17] Welling DB, Barnes DE. Acoustic stimulation of the semicircular canals. Otolaryngol Clin North Am 1995;28:207–19. 7739866

[18] Devèze A, Koka K, Tringali S, Jenkins HA, Tollin DJ. Active middle ear implant application in case of stapes fixation: a temporal bone study. Otol Neurotol 2010;31:1027–34. 10.1097/MAO.0b013e3181edb6d1 20679957

[19] Mattingly JK, Greene NT, Jenkins HA, Tollin DJ, Easter JR, Cass SP. Effects of Skin Thickness on Cochlear Input Signal Using Transcutaneous Bone Conduction Implants. Otol Neurotol 2015 10.1097/MAO.0000000000000814 PMC4537381 26164446

[20] ASTM. International F2504-05: Standard Practice for Describing System Output of Implantable Middle Ear Hearing Devices ASTM, Philadelphia 2005.

[21] Verhaert N, Hofmann M, Wouters J. Transient and Steady State Auditory Responses With Direct Acoustic Cochlear Stimulation. Ear Hear 2015;36:320–9. 10.1097/AUD.0000000000000117 25401379

[22] Stieger C, Rosowski JJ, Nakajima HH. Comparison of forward (ear-canal) and reverse (round-window) sound stimulation of the cochlea. Hear Res 2013;301:105–14. 10.1016/j.heares.2012.11.005 23159918 PMC3584235

[23] Stenfelt S, Hato N, Goode RL. Fluid volume displacement at the oval and round windows with air and bone conduction stimulation. J Acoust Soc Am 2004;115:797–812. 10.1121/1.1639903 15000191

[24] Stenfelt S, Hato N, Goode RL. Round window membrane motion with air conduction and bone conduction stimulation. Hear Res 2004;198:10–24. 10.1016/j.heares.2004.07.008 15567598

[25] Asai M, Huber AM, Goode RL. Analysis of the best site on the stapes footplate for ossicular chain reconstruction. Acta Otolaryngol 1999;119:356–61. 10.1080/00016489950181396 10380743

[26] Grossöhmichen M, Salcher R, Kreipe H-H, Lenarz T, Maier H. The Codacs^TM^ Direct Acoustic Cochlear Implant Actuator: Exploring Alternative Stimulation Sites and Their Stimulation Efficiency. PLoS One 2015;10:e0119601 10.1371/journal.pone.0119601 25785860 PMC4364953

[27] Gostian A, Pazen D, Ortmann M, Luers J, Anagiotos A, Hu K, et al. Impact of Coupling Techniques of an Active Middle Ear Device to the Round Window Membrane for the Backward Stimulation of the Cochlea 2014:111–7. 10.1097/MAO.0000000000000655 25406868

[28] Rosowski JJ, Chien W, Ravicz ME, Merchant SN. Testing a method for quantifying the output of implantable middle ear hearing devices. Audiol Neurootol 2007;12:265–76. 10.1159/000101474 17406105 PMC2596735

[29] Chatzimichalis M, Sim JH, Huber AM. Assessment of a direct acoustic cochlear stimulator. Audiol Neurootol 2012;17:299–308. 10.1159/000339214 22739432

[30] Shambaugh GE, Wiet RJ. The fenestration operation in 1979. Am J Otol 1979;1:1–6. 554464

[31] Farrior JB, Rophie SS. Fenestration of the horizontal semicircular canal in congenital conductive deafness. Laryngoscope 1985;95:1029–36. 4033323

[32] Nakajima HH, Ravicz ME, Merchant SN, Peake WT, Rosowski JJ. Experimental ossicular fixations and the middle ear’s response to sound: evidence for a flexible ossicular chain. Hear Res 2005;204:60–77. 10.1016/j.heares.2005.01.002 15925192

[33] Rosnow RL, Rosenthal R. Beginning behavioural research: a conceptual primer 5th ed. Englewood Cliffs, NJ: Pearson/Prentice Hall; 2005.

[34] Whittemore KR, Merchant SN, Poon BB, Rosowski JJ. A normative study of tympanic membrane motion in humans using a laser Doppler vibrometer (LDV). Hear Res 2004;187:85–104. 14698090 10.1016/s0378-5955(03)00332-0

[35] Tonndorf J. Bone conduction. Studies in experimental animals. Acta Otolaryngol 1966:Suppl 213:1+. 5934763

[36] Agrawal SK, Parnes LS. Human experience with canal plugging. Ann N Y Acad Sci 2001;942:300–5. 11710471 10.1111/j.1749-6632.2001.tb03754.x

[37] Ramakrishna J, Goebel JA, Parnes LS. Efficacy and safety of bilateral posterior canal occlusion in patients with refractory benign paroxysmal positional vertigo: case report series. Otol Neurotol 2012;33:640–2. 10.1097/MAO.0b013e31824bae56 22429946

[38] Charpiot A, Rohmer D, Gentine A. Lateral semicircular canal plugging in severe Ménière’s disease: a clinical prospective study about 28 patients. Otol Neurotol 2010;31:237–40. 10.1097/MAO.0b013e3181ca85a2 20101162

[39] Maier H, Salcher R, Schwab B, Lenarz T. The effect of static force on round window stimulation with the direct acoustic cochlea stimulator. Hear Res 2013;301:115–24. 10.1016/j.heares.2012.12.010 23276731

[40] Bernhard H, Stieger C, Perriard Y. Design of a semi-implantable hearing device for direct acoustic cochlear stimulation. IEEE Trans Biomed Eng 2011;58:420–8. 10.1109/TBME.2010.2087756 20959263

[41] Pennings RJE, Ho A, Brown J, van Wijhe RG, Bance M. Analysis of Vibrant Soundbridge placement against the round window membrane in a human cadaveric temporal bone model. Otol Neurotol 2010;31:998–1003. 10.1097/MAO.0b013e3181e8fc21 20601915

[42] Stenfelt S. Inner ear contribution to bone conduction hearing in the human. Hear Res 2014;329:41–51. 10.1016/j.heares.2014.12.003 25528492

[43] Pisano D V, Niesten MEF, Merchant SN, Nakajima HH. The effect of superior semicircular canal dehiscence on intracochlear sound pressures. Audiol Neurootol 2012;17:338–48. 000339653. 10.1159/000339653 22814034 PMC3541532

[44] Merchant SN, Rosowski JJ. Conductive hearing loss caused by third-window lesions of the inner ear. Otol Neurotol 2008;29:282–9. 10.1097/mao.0b013e318161ab24 18223508 PMC2577191

[45] Wever EG, Lawrence M, Smith KR. The middle ear in sound conduction. Arch Otolaryngol 1949;48:19–35. 18113037 10.1001/archotol.1948.00690040026003

[46] Olson ES. Direct measurement of intra-cochlear pressure waves. Nature 1999;402:526–9. 10.1038/990092 10591211

[47] Nakajima HH, Dong W, Olson ES, Merchant SN, Ravicz ME, Rosowski JJ. Differential intracochlear sound pressure measurements in normal human temporal bones. J Assoc Res Otolaryngol 2009;10:23–36. 10.1007/s10162-008-0150-y 19067078 PMC2644388

[48] Lempert J. Physiology of hearing; what have we learned about it following fenestration surgery? AMA Arch Otolaryngol 1952;56:101–13. 14943329 10.1001/archotol.1952.00710020120001

[49] Kitahara T, Kamakura T, Ohta Y, Morihana T, Horii A, Uno A, et al Chronic Otitis Media With Cholesteatoma With Canal Fistula and Bone Conduction Threshold After Tympanoplasty With Mastoidectomy. Otol Neurotol 2014;35:981–8. 10.1097/MAO.0000000000000306 24936778

[50] Coulson CJ, Assadi MZ, Taylor RP, Du X, Brett PN, Reid AP, et al A smart micro-drill for cochleostomy formation: a comparison of cochlear disturbances with manual drilling and a human trial. Cochlear Implants Int 2013;14:98–106. 10.1179/1754762811Y.0000000018 22333534

